# Association Between Intraoperative Arterial Oxygen Tension and Postoperative Opioid Consumption: A Large-Scale Cohort Study Using Overlap Weighting Analysis

**DOI:** 10.3390/medicina62071318

**Published:** 2026-07-08

**Authors:** Moon Ok Lee, Hanna Koh, Sung-Ae Cho

**Affiliations:** Department of Anesthesiology and Pain Medicine, Samsung Changwon Hospital, Sungkyunkwan University School of Medicine, 158 Paryong-ro, Masanhoewon-gu, Changwon-si 51353, Gyeongsangnam-do, Republic of Korea; lmo602@hanmail.net (M.O.L.); na1130@gmail.com (H.K.)

**Keywords:** hyperoxia, analgesics, opioid, pain, postoperative, postoperative complications, lung diseases, blood gas analysis

## Abstract

*Background and Objectives*: The clinical impact of hyperoxia, particularly its analgesic effects and potential pulmonary risks, remains controversial. This study investigated whether higher intraoperative arterial oxygen tension (PaO_2_) reduces postoperative analgesic requirements and its association with postoperative pulmonary complications (PPCs). *Materials and Methods*: This retrospective cohort study included 1194 patients undergoing non-cardiac surgery. Patients were categorized into lower PaO_2_ (PaO_2_ ≤ 200 mmHg) and higher PaO_2_ (PaO_2_ > 200 mmHg) groups based on intraoperative arterial blood gas analysis. Overlap weighting was used to balance baseline characteristics and intraoperative variables. Primary outcomes included postoperative opioid consumption (measured in morphine milligram equivalents, MME) at 0–24 and 24–48 h. Secondary outcomes included pain intensity, frequency of rescue analgesics, the incidence of PPCs and in-hospital mortality. *Results:* After overlap weighting, the higher PaO_2_ group showed significantly lower strong opioid consumption during the 24–48 h period compared to the lower PaO_2_ group (0.22 ± 0.04 vs. 0.50 ± 0.11 MME; adjusted *p* = 0.014). The frequency of rescue analgesics was significantly reduced in the higher PaO_2_ group (1.04 ± 0.06 vs. 1.35 ± 0.11; adjusted *p* = 0.007). However, pain intensity and the total MME for 0–24 h did not differ. There was no significant difference in the incidence of total PPCs or total adverse events between the two groups. *Conclusions*: Higher intraoperative PaO_2_ was associated with a significant decrease in opioid requirements during the postoperative 24–48 h and frequency of rescue analgesics without increasing PPCs and adverse events. These findings support the need for prospective studies to determine whether higher intraoperative PaO_2_ may have a clinically meaningful role in perioperative analgesic strategies.

## 1. Introduction

The role of intraoperative hyperoxia in perioperative pain management remains a subject of debate. While hyperoxia has been hypothesized to modulate central pain pathways and increase endogenous opioid secretion, clinical evidence remains inconsistent [1]. A large randomized controlled trial found that 80% inspired oxygen fraction (FiO_2_) did not reduce postoperative pain scores or opioid consumption [2]. However, these negative findings may reflect methodological limitations of using FiO_2_ as the sole indicator of oxygen exposure rather than actual tissue oxygenation [2,3].

High arterial oxygen tension (PaO_2_) is known to induce absorption atelectasis and postoperative pulmonary complications (PPCs) through oxygen toxicity and denitrogenation. Despite this, studies simultaneously assessing analgesic efficacy and pulmonary safety remain rare, resulting in inconsistent risk–benefit findings [2,3,4]. This discrepancy may be due primarily to study designs that do not comprehensively consider pain control and pulmonary risk, and that assess these important parameters independently rather than as a combined risk–benefit profile. Furthermore, FiO_2_, which does not adequately reflect actual tissue oxygen delivery, is often used as a surrogate measure [2,3]. Relying on fraction of inspired oxygen as a surrogate indicator of oxygen exposure overlooks interindividual variability in arterial oxygenation and tissue oxygen delivery, which are more directly related to pain control and lung injury [3,5,6].

Therefore, in this study, we defined higher intraoperative PaO_2_ as a PaO_2_ measurement exceeding 200 mmHg and simultaneously assessed postoperative potent opioid use and PPCs. Using overlapping weighting to minimize confounding factors, we aimed to determine whether higher PaO_2_ could reduce opioid use without compromising pulmonary safety.

## 2. Materials and Methods

### 2.1. Study Design and Setting

This study was approved by the Institutional Review Board (IRB) of our hospital (Approval No. SCMC IRB 2025-12-014]). Due to the retrospective nature of the study, informed consent was waived. We screened adult patients aged 18 years or older who underwent non-cardiac surgery under general anesthesia from June 2023 to May 2025 and underwent at least one intraoperative arterial blood gas analysis (ABGA) performed at least 30 min after the surgical incision. Patients who underwent cardiac surgery, had chronic opioid use, had an opioid allergy, or required cardiopulmonary resuscitation or extracorporeal membrane oxygenation (ECMO) during surgery were excluded from the study. To ensure stable baseline status, patients with active lung disease before surgery were also excluded. Active lung disease requiring exclusion was defined as acute pneumonia, active tuberculosis, pulmonary edema, or symptomatic pleural effusion. Preexisting stable, non-acute pulmonary conditions were systematically categorized into the five balanced covariates as defined in Section 2.2. Additionally, patients who received preoperative oxygen supplementation or had missing data on intraoperative ABGA or postoperative analgesic administration were excluded from the analysis.

### 2.2. Data Collection and Variables

Data were extracted from the electronic medical record (EMR) system in January 2026 following the IRB approval. Baseline variables included demographic information, body mass index (BMI), American Society of Anesthesiologists (ASA) physical status, and major comorbidities (cardiovascular, pulmonary, renal, hepatic, metabolic, and malignant disease). To specifically consider preoperative lung conditions, non-acute findings were classified into five categories: (1) chronic obstructive/structural disease (e.g., chronic obstructive pulmonary disease (COPD), emphysema, bronchiectasis), (2) asthma, (3) inactive tuberculosis scars, (4) interstitial or restrictive lung disease, and (5) minor structural abnormalities (including pleural thickening and calcified nodules). Intraoperative data included the type and time of surgery and anesthesia, surgical position and department, intraoperative opioid dosage, use of PCA and nerve blocks, and use of vasopressors. All intraoperative ABGA results (pH, PaCO_2_, PaO_2_, HCO_3_^−^) were extracted.

### 2.3. Exposure and Group Definition

The primary exposure factor was defined as PaO_2_ during surgery. Patients with PAO_2_ exceeding 200 mmHg on at least one recorded test during the surgery were classified into the higher PaO_2_ group. This threshold was chosen to indicate significant hyperoxia, based on previous studies that defined PaO_2_ > 200 mmHg as severe hyperoxia and that found that high PaO_2_ is associated with denitrogenation and absorption atelectasis during anesthesia [3,7]. Conversely, patients whose PaO_2_ values remained consistently below 200 mmHg throughout the surgery were assigned to the lower PaO_2_ group. For exploratory sensitivity analyses, higher PaO_2_ intensities were further stratified into moderately higher PaO_2_ (200 ≤ PAO_2_ ≤ 300 mmHg) and severely higher PaO_2_ (PAO_2_ > 300 mmHg).

### 2.4. Outcome Measures

Intraoperative analgesia followed institutional standards, with intravenous fentanyl or remifentanil titrated to hemodynamic responses and additional boluses of morphine, pethidine or fentanyl administered at the anesthesiologist’s discretion. Regional and neuraxial techniques, including peripheral nerve blocks and epidural analgesia, were recorded and modeled as a binary “nerve block” variable.

Postoperative analgesia was based on a standard intravenous PCA regimen (fentanyl) and additional analgesia according to institutional protocols using either supplemental opioids or non-opioid agents such as NSAIDs and acetaminophen. Although PCA prescription parameters were documented, the electronic record did not provide sufficiently granular data on cumulative infusion volumes to support accurate calculation of PCA-delivered MME. We therefore focused our primary outcome on strong opioids administered outside of PCA, representing clinician-driven supplemental analgesia beyond the background regimen.

The primary outcome was supplemental strong opioid consumption (excluding PCA-delivered doses) during the first 48 postoperative hours, converted to IV morphine milligram equivalents (MME). Postoperative opioids were classified as strong (morphine, pethidine, fentanyl, oxycodone) or weak (tramadol) to assess differential effects on high-potency μ-agonists. IV morphine equivalents were calculated using published equianalgesic conversion tables and palliative care opioid conversion guidelines (8–10). Based on these sources, we assumed that 1 μg of intravenous fentanyl, 1 mg of intravenous meperidine (pethidine), and 1 mg of intravenous tramadol were equivalent to approximately 0.066 mg, 0.13 mg, and 0.10 mg of intravenous morphine, respectively, and that 1 mg of intravenous oxycodone was approximately equivalent to 1.5 mg of intravenous morphine [8]. For transdermal fentanyl, we used standard conversions in which a 25 μg/hour patch is considered equivalent to 60 mg/day of oral morphine (approximately 20 mg/day of intravenous morphine), with other patch strengths scaled proportionally [9,10]. For oral oxycodone-containing products, we assumed that 10 mg of oral oxycodone is approximately equivalent to 15 mg of oral morphine (about 5 mg of intravenous morphine) and calculated morphine equivalents accordingly [9,10].

Secondary outcome variables included consumption of strong opioids (converted to MME) and a weak opioid (tramadol) in two time intervals (0–24 h and 24–48 h), consumption of acetaminophen and NSAIDs over the 0–48 h period, frequency of rescue analgesic administration over the 48-h period, and pain intensity assessed using the numeric rating score (NRS, 0–10). A rescue analgesic in this context was defined as any additional analgesic administered in response to inadequate pain control despite ongoing PCA, including supplemental strong opioids, tramadol, NSAIDs, and acetaminophen. NRSs were summarized as the highest value during 0–24 h, 24–48 h, and 0–48 h. Patients who were not evaluable were excluded from the NRS analysis.

PPCs were evaluated as a composite outcome (respiratory infection, failure, pleural effusion, atelectasis, pneumothorax, bronchospasm, aspiration pneumonitis) during hospitalization, with incidences reported both within 48 h and overall. Other safety outcomes included in-hospital mortality, intensive care unit (ICU) admission, and a composite of major non-respiratory adverse events (cardiovascular, surgical, infectious, neurological, and multiorgan failure). PPCs were identified retrospectively from the EMR using predefined criteria based on clinical diagnoses and radiologic findings documented by treating physicians. Respiratory infection was defined as a new diagnosis of pneumonia or lower respiratory tract infection requiring antibiotic therapy. Respiratory failure was defined as the need for non-invasive or invasive ventilatory support, or persistent postoperative hypoxemia requiring high-flow oxygen therapy. Pleural effusion and pneumothorax were recorded when documented in radiology reports as new postoperative findings. Atelectasis was defined as a new radiologic report of segmental, lobar, or more extensive collapse or volume loss compatible with atelectasis on chest radiography or computed tomography during the index hospitalization. Bronchospasm and aspiration pneumonitis were defined based on clinical documentation by the attending physicians. Postoperative chest imaging (radiography or CT) was performed at the discretion of the treating team, usually in response to respiratory symptoms, hypoxemia, or suspected infection, rather than as routine screening for all patients. In addition, we evaluated a more clinically anchored composite of moderate-to-severe PPCs, including postoperative respiratory failure requiring non-invasive or invasive ventilatory support, pneumonia requiring antibiotic treatment, and pleural effusion or pneumothorax requiring procedural intervention. This composite was analyzed in the overlap-weighted cohort using the same modeling approach as for the PPC outcome.

### 2.5. Statistical Analysis

Propensity scores were estimated using multivariable logistic regression including demographics, BMI, all comorbidities (including chronic lung disease classified into five categories), ASA status, surgical and anesthetic factors (surgery time, emergency status, surgical position, surgery department, type of anesthesia), and analgesic-related variables (use of intraoperative opioid, PCA use, nerve block). Overlap weighting based on the propensity score was applied to achieve covariate balance (target standardized mean differences [SMDs] < 0.1) and to estimate the average treatment effect in the overlap population. This procedure assigns each patient a weight proportional to the probability of receiving the opposite treatment, and then re-scales the weights so that the total weight is the same in the two groups. As a result, the analysis is conducted in a weighted pseudo-population in which baseline covariates are well balanced between the lower PaO_2_ and higher PaO_2_ groups. The “effective N” reported for the overlap-weighted cohort represents the sum of the overlap weights in each group rather than the raw number of patients, and reflects the information content of the weighted pseudo-population used in the regression models [11].

Continuous variables were compared using weighted *t*-tests, and categorical variables were analyzed using weighted Rao–Scott chi-square tests. Subgroup analyses for strong opioid consumption were performed according to predefined stratification variables (age, sex, cancer history, emergency status) and reported as beta coefficients with 95% confidence intervals.

Multivariable overlap-weighted linear regression models were used to determine the independent effect of higher PaO_2_ on postoperative strong opioid consumption. The primary outcome model was adjusted for demographic variables (age, sex, BMI), ASA physical status, surgical factors (operating department, operative time, emergency status), and an extensive list of comorbidities, including hypertension, diabetes mellitus, dyslipidemia, arrhythmia, angina or myocardial infarction, stroke, chronic kidney disease, chronic liver disease, cancer, and five predefined chronic lung disease categories. Analgesic variables such as use of intraoperative opioid, PCA use, and nerve block were also included as covariates.

Exploratory sensitivity analyses further stratified patients by maximal intraoperative PaO_2_ (≤200, 200–300, >300 mmHg) to evaluate potential trends. For less frequent safety outcomes, additional comparisons were performed in the unweighted cohort using Fisher’s exact test. All statistical analyses were performed using R software (version 4.4.1, R Foundation for Statistical Computing, Vienna, Austria), and a *p* value of <0.05 was considered statistically significant.

## 3. Results

### 3.1. Patient Characteristics and Overlap Weighting

**Figure 1:**
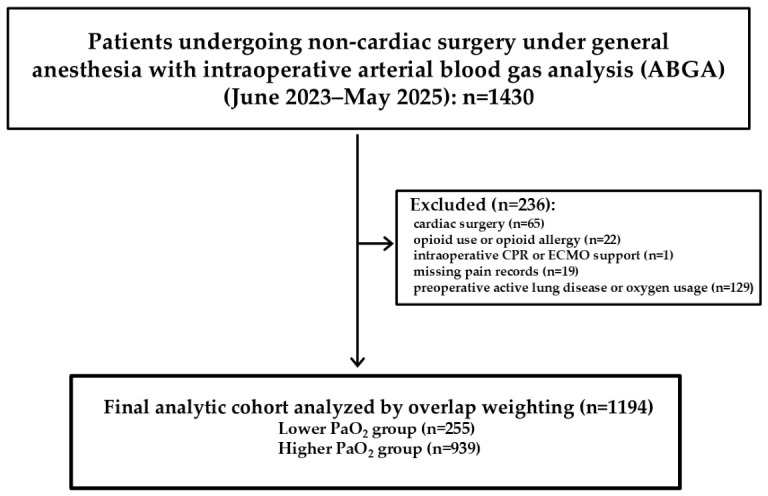
Flowchart of patient selection. Overlap weighting was applied to the entire eligible population (*n* = 1194) without omitting patients, so the sample size does not decrease further in the final analytic step.

Of the 1430 patients screened, 1194 were included in the study (255 in the lower PaO_2_ group and 939 in the higher PaO_2_ group, Figure 1). Before weighting, the two groups differed in BMI, cancer history, and preoperative opioid use. After applying overlapping weighting, all baseline and intraoperative variables were well balanced (all SMD < 0.1, Table 1). Regarding parameters related to exposure capturing, the timing of the first intraoperative ABGA sampling after surgical incision was comparable between the cohorts (49.73 ± 13.43 min for the lower PaO_2_ group vs. 50.38 ± 14.77 min for the higher PaO_2_ group; adjusted *p* = 0.526, SMD = 0.046). In contrast, the mean number of intraoperative ABGAs per patient was higher in the higher PaO_2_ group than in the lower PaO_2_ group (1.93 ± 1.31 vs. 1.61 ± 0.90; adjusted *p* < 0.001, SMD = 0.279).

### 3.2. Postoperative Opioid Consumption and Pain Intensity

Postoperative supplemental strong opioid requirements excluding PCA-delivered doses, as the primary outcome, are presented in Table 2. Strong opioid consumption between 24 and 48 h was significantly lower in the higher PaO_2_ group than in the lower PaO_2_ group (0.22 ± 0.04 vs. 0.50 ± 0.11 MME; adjusted *p* = 0.014), and the frequency of rescue analgesic administrations was also reduced (1.04 ± 0.06 vs. 1.35 ± 0.11; *p* = 0.007). In contrast, weak opioid consumption and total MME between 0 and 24 h did not differ significantly between the two groups. NRS pain scores between 0 and 24 h, and between 24 and 48 h, as well as the maximal NRS, were similar between the two groups.

### 3.3. Multivariable Weighted Regression Analysis for Opioid Requirements

**Figure 2:**
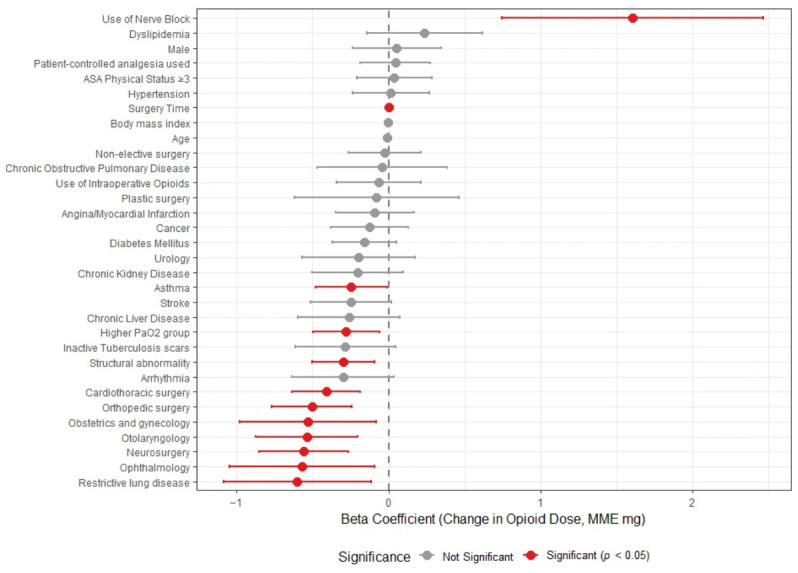
Factors associated with postoperative strong opioid consumption (24–48 h). Beta coefficients and 95% confidence intervals are derived from multivariable overlap-weighted linear regression models adjusted for age, sex, BMI, ASA physical status, surgical department, surgery time, emergency status, PCA use, nerve block, intraoperative opioid use, and comorbidities.

In the primary overlap-weighted multivariable linear regression model (Figure 2), higher intraoperative PaO_2_ was significantly associated with reduced strong opioid consumption during the 24–48 h postoperative period (adjusted estimate −0.278 MME; 95% CI −0.499 to −0.058; *p* = 0.014), after adjustment for age, sex, BMI, ASA physical status, surgery time, non-emergency surgery status, use of intraoperative opioids, patient-controlled analgesia use, nerve block, surgical department, and comorbidities. Several covariates also showed statistically significant associations with 24–48 h strong opioid requirements, including asthma, chronic kidney disease, chronic liver disease, restrictive lung disease, and orthopedic, obstetric–gynecologic, otolaryngologic, neurosurgical, and ophthalmologic surgical departments, as well as nerve block use and dyslipidemia. Other adjusted coefficients were not statistically significant.

### 3.4. Subgroup Analyses

**Figure 3:**
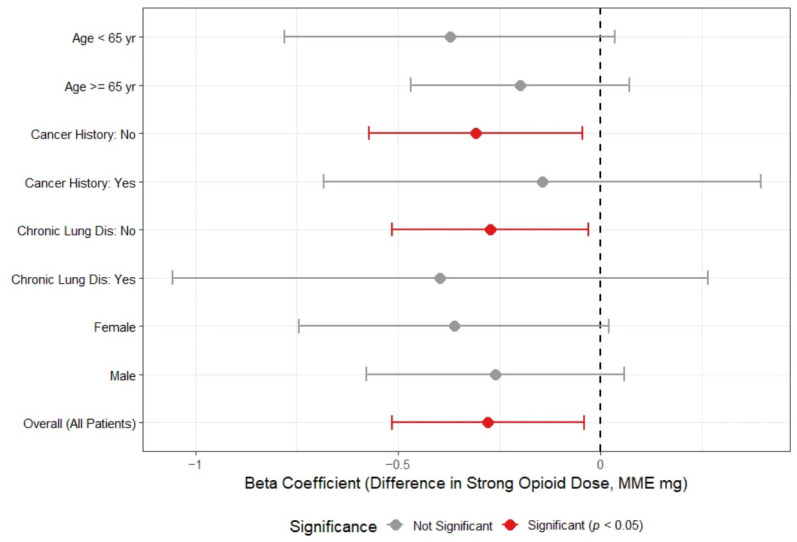
Subgroup analysis of the effect of higher PaO_2_ on strong opioid consumption.

The adjusted estimates for reduced opioid consumption were consistently negative in all subgroups (Figure 3). Specifically, in the overall subgroup model adjusted for age, sex, BMI, ASA class, and cancer only, higher PaO_2_ was associated with a reduction in strong opioid consumption (adjusted estimate: −0.278; 95% CI: −0.515 to −0.041; *p* = 0.021). Statistically significant reductions were likewise observed in patients without a history of cancer (adjusted estimate: −0.308; 95% CI: −0.571 to −0.045; *p* = 0.022), and those without chronic lung disease (adjusted estimate: −0.272; 95% CI: −0.515 to −0.029; *p* = 0.028) in these subgroup-specific overlap-weighted models.

### 3.5. Postoperative Pulmonary Complications and Safety Outcomes

Postoperative pulmonary complications and safety outcomes are presented in Table 3. There were no significant differences in the incidences of PPCs or major adverse events within 48 h postoperatively. The incidence of total PPCs was similar between the higher PaO_2_ and lower PaO_2_ groups (27.5% vs. 26.5%, respectively; adjusted *p* = 0.767). When a more clinically anchored composite of moderate-to-severe PPCs was used (respiratory failure requiring ventilatory support, pneumonia requiring antibiotic therapy, and pleural effusion or pneumothorax requiring intervention), the incidence remained similar between the lower PaO_2_ and higher PaO_2_ groups, and no significant differences were observed in the overlap-weighted analyses. Additionally, the incidence of total adverse events did not differ significantly between the two groups, with 8.4% in the higher PaO_2_ group and 10.2% in the lower PaO_2_ group (adjusted *p* = 0.381). Other clinical outcomes, including in-hospital mortality, were similar between the two groups.

### 3.6. Exploratory Sensitivity and Maximal Intensity Analysis

In an exploratory sensitivity analysis stratified by the maximum intraoperative PaO_2_ (Table 4), an association between a shift toward moderately to severely higher PaO_2_ and reduced late strong opioid consumption was evaluated. Compared with the overall cohort average (0.36 ± 0.06 MME), the higher PaO_2_ group required significantly lower doses of opioids (0.22 ± 0.04 MME), whereas the lower PaO_2_ group exceeded the cohort average (0.50 ± 0.11 MME). The requirement, which was 0.50 ± 0.11 MME in the lower PaO_2_ group, decreased to 0.23 ± 0.04 MME and 0.20 ± 0.09 MME in the moderate and hyperoxic groups, respectively (P-trend = 0.014). In addition, a sequential analysis of maximal PaO_2_ revealed that for every 100 mmHg increase in maximal PaO_2_, opioid dose decreased by 0.128 MME (*p* = 0.018). In contrast, the incidences of total PPCs (26.5% vs. 26.9% vs. 30.3%; weighted Rao–Scott *p* = 0.751) and total adverse events (10.2% vs. 8.4% vs. 8.1%; weighted Rao–Scott *p* = 0.556) were not significantly different by PaO_2_ interval.

## 4. Discussion

The objective of this study was to investigate the effects of higher PaO_2_ during the intraoperative period, defined by PaO_2_ > 200 mmHg, on postoperative opioid consumption and pulmonary outcomes. In this retrospective cohort, higher PaO_2_ was associated with a statistically significant but small reduction in supplemental strong opioid use during the 24–48 h postoperative period, without an observed increase in PPCs or other major adverse events.

Previous studies have shown that intraoperative administration of 80% oxygen does not reduce postoperative pain or postoperative opioid consumption and is not associated with an increased risk of PPCs compared with 30% oxygen in patients undergoing colon surgery [2,12]. However, rather than simply increasing FiO_2_, our study analyzed patients with an actual partial pressure of blood oxygen exceeding 200 mmHg using ABGA, thereby assessing the analgesic effect associated with improved tissue oxygenation. Also, recent studies using hyperbaric oxygen therapy have confirmed the benefits of oxygen [13,14]. However, whereas previous studies focused on reducing muscle damage and inflammation or managing postoperative complications with hyperbaric oxygen therapy, our study examined changes in opioid consumption in the real world of the operating room.

The physiological basis for the observed analgesic effect of higher PaO_2_ remains unmeasured in our study, though the prior literature suggests several speculative pathways. First, higher PaO_2_ has been hypothesized to modulate local inflammatory responses by increasing tissue oxygen tension. Surgical wounds are usually in a hypoxic and acidic environment, conditions that lead to incision pain and inflammation. Improved oxygen supply supports aerobic metabolism and collagen synthesis, promoting wound healing and suppressing inflammatory responses [15,16]. Experimental studies on nitroxidative signaling have shown that oxidative and inflammatory mediators influence nociceptor sensitivity and pain transmission [17]. Second, oxygen is essential for collagen synthesis and wound healing, and high tissue oxygen tension has been associated with increased collagen accumulation and maturation in surgical and experimental wounds [15]. Improved oxygenation may indirectly reduce peripheral sensitization at the incision site by promoting more efficient wound healing [18]. Additionally, appropriate levels of reactive oxygen species (ROS) generated under hyperoxic conditions may act as signaling molecules that regulate pain transmission. Nitroxidative signaling plays a complex role in pain pathways, with oxygen and nitrogen species contributing to both the generation and resolution of inflammatory and neuropathic pain, depending on context and concentration [17]. Therefore, it is possible to hypothesize that changes in redox signaling induced by changes in oxygen tension may modulate pain processing. Because this mechanism remains only a hypothesis in the perioperative setting, direct validation of these pathways will require mechanistic studies with biomarker collection.

Another interesting finding from our study is that the difference in opioid requirements was more pronounced in the 24–48 h period than in the immediate postoperative period (0–24 h). This temporal pattern is more compatible with the influence of intraoperative oxygenation on time-dependent biological processes such as inflammation regulation, wound oxygenation, and tissue repair, rather than a purely immediate neurophysiological effect [18]. In a multivariable weighted linear regression model, higher intraoperative PaO_2_ was identified as a significant independent factor associated with reduced strong opioid consumption during the late postoperative period (adjusted estimate: −0.278; 95% CI: −0.499 to −0.058; adjusted *p* = 0.014; Figure 2). Furthermore, subgroup analyses confirmed consistent opioid savings across most predefined strata, including patients without cancer (adjusted estimate: −0.308; *p* = 0.022) or chronic lung disease (adjusted estimate: −0.272; *p* = 0.028; Figure 3). Consistent with these results, an exploratory sensitivity analysis stratified by maximal intraoperative PaO_2_ values revealed that strong opioid consumption during the 24–48 h postoperative period progressively decreased from lower PaO_2_ to higher PaO_2_ (moderate and severe). Together, these findings are compatible with a graded association between intraoperative PaO_2_ and postoperative opioid requirements and raise the hypothesis that intraoperative oxygenation may modulate postoperative opioid requirements through time-dependent biological processes [18]. However, because PaO_2_ categories were derived from intermittent, non-standardized ABGA measurements and did not account for temporal exposure, these results should be interpreted with caution, requiring confirmation in prospective studies with more granular oxygenation monitoring.

Although NRSs were similar between groups at all time points, the higher PaO_2_ group required significantly lower opioid doses to maintain this level of analgesia. The lack of a significant difference in postoperative pain scores, despite a modest reduction in opioid consumption, likely reflects the clinical nature of reactive analgesic titration. Because ward clinicians typically titrate rescue opioids dynamically to maintain a uniform target level of patient comfort, this observed discrepancy suggests that patients in the higher PaO group than those who remained strictly normoxic throughout surgery. Furthermore, intraoperative FiO_2_ and PaO_2_ data are not routinely communicated to or reviewed by postoperative ward teams, making it unlikely that differential prescribing behavior systematically drove the observed difference in opioid consumption, although this possibility cannot be formally excluded given the retrospective design. Instead, this may imply that higher intraoperative PaO_2_ contributes to the effectiveness of pain management and lowers the threshold for pharmacological intervention. As is well known, reducing systemic opioid exposure is clinically beneficial, as high doses are associated with an increased incidence of opioid-related adverse effects, such as postoperative nausea and vomiting, constipation, and respiratory depression [19,20,21]. Our finding of reduced opioid use during higher PaO_2_ suggests that strategies for achieving adequate analgesia with relatively low opioid doses may contribute to reducing these complications and improving patient safety [21].

Perioperative oxygen therapy raises concerns about absorption atelectasis and oxygen toxicity. However, in this study, there was no significant difference in the overall PPC incidence between the higher PaO_2_ (27.5%) and the lower PaO_2_ groups (26.5%). These findings suggest that, within the exposure range captured by our intermittent ABGA measurements, higher intraoperative PaO_2_ was not associated with an increased incidence of clinically apparent PPCs. However, because we did not quantify the cumulative duration or area under the curve of hyperoxic exposure, which is important for pulmonary toxicity, our data cannot exclude pulmonary toxicity with more prolonged or extreme oxygen administration [22,23,24,25]. Taken together, our data indicate that, in this cohort, higher intraoperative PaO_2_ was associated with lower postoperative opioid requirements and was not associated with an observable increase in PPCs. It is plausible that the biological threshold for modulating inflammation and wound-related nociception is lower than the cumulative dose required to trigger overt pulmonary oxygen toxicity, yet defining these thresholds will require prospective, exposure-controlled studies.

This study has several limitations. First, despite overlap weighting, residual confounding from unmeasured perioperative variables remains possible. Our database did not capture intraoperative fluid volumes, blood loss, transfusion requirements, surgical trauma severity, or postoperative ward analgesic protocols, and postoperative opioid prescribing practices may have varied across surgical departments. Because this retrospective design precluded formal caregiver blinding, these non-standardized departmental analgesic protocols could still contribute to residual variability in postoperative opioid administration. Second, intraoperative oxygen exposure was assessed using intermittent ABGA measurements rather than continuous monitoring, so we could not quantify the duration or area under the curve of PaO_2_ above specific thresholds. Patients whose intraoperative PaO_2_ remained below 200 mmHg may also represent a physiologically distinct subgroup (e.g., higher V/Q mismatch or greater surgical complexity), introducing potential confounding by indication. Accordingly, the trends stratified by maximal intraoperative PaO_2_ should be interpreted as exploratory, and causal inference is not warranted. In addition, the subgroup analyses were exploratory because of the modest effective sample size of the weighted cohort and the multiple subgroups examined. Third, because cumulative PCA infusion volumes were not reliably recorded in the EMR, we could not reconstruct PCA-delivered opioid doses. Our opioid outcomes therefore reflect only strong opioid doses administered in addition to background PCA regimens, and residual differences in PCA settings or infused volumes may still have influenced postoperative opioid requirements. Finally, our inclusion criterion of at least one intraoperative ABGA measurement inherently selected patients undergoing major, complex, or prolonged procedures requiring arterial monitoring. As a result, our findings may not be generalizable to lower-risk surgical populations undergoing minor procedures without routine ABGA. Therefore, these findings demonstrate a statistical association rather than providing clinical evidence to support the routine induction of intraoperative hyperoxia in standard anesthetic practice.

## 5. Conclusions

Higher intraoperative PaO_2_ exceeding PaO_2_ 200 mmHg was associated with a small reduction in supplemental strong opioid requirements during 24–48 h postoperatively, without an accompanying increase in pulmonary complications or other adverse events. Although the absolute effect size was modest and pain scores were similar between groups, the consistent direction of association across subgroups and sensitivity analyses is consistent with biologically plausible mechanisms by which intraoperative PaO_2_ might affect postoperative opioid requirements. These findings support the need for prospective studies with detailed oxygen exposure metrics to determine whether intraoperative PaO_2_ has a clinically meaningful role in perioperative analgesic strategies.

## Figures and Tables

**Table 1:** Baseline characteristics before and after overlap weighting.

Variables		Before Weighting (*n* = 1194)			After Overlap Weighting (Effective *n* = 360.6)		
		Lower PaO_2_ (*n* = 255)	Higher PaO_2_ (*n* = 939)	SMD	Lower PaO_2_ (*n* = 180.3)	Higher PaO_2_ (*n* = 180.3)	SMD
Demographics							
Male, n (%)		142 (55.7)	531 (56.5)	0.017	100.0 (55.4)	100.0 (55.4)	<0.001
Age (years)		63.26 (13.02)	64.28 (12.86)	0.079	63.63 (12.97)	63.63 (13.50)	<0.001
BMI (kg/m^2^)		25.14 (4.80)	23.86 (3.78)	0.296	24.63 (4.30)	24.63 (4.05)	<0.001
ASA Physical Status ≥ 3		133 (52.2)	516 (55.0)	0.056	95.1 (52.7)	95.1 (52.7)	<0.001
Comorbidities, n (%)							
Hypertension		114 (44.7)	435 (46.3)	0.033	80.8 (44.8)	80.8 (44.8)	<0.001
Diabetes Mellitus		64 (25.1)	268 (28.5)	0.078	46.4 (25.7)	46.4 (25.7)	<0.001
Dyslipidemia		38 (14.9)	142 (15.1)	0.006	26.8 (14.9)	26.8 (14.9)	<0.001
Arrhythmia		6 (2.4)	28 (3.0)	0.039	4.7 (2.6)	4.7 (2.6)	<0.001
Angina/MI		14 (5.5)	84 (8.9)	0.134	11.0 (6.1)	11.0 (6.1)	<0.001
Stroke		10 (3.9)	57 (6.1)	0.099	8.3 (4.6)	8.3 (4.6)	<0.001
CKD		3 (1.2)	13 (1.4)	0.018	2.4 (1.3)	2.4 (1.3)	<0.001
Chronic Liver Disease		19 (7.5)	74 (7.9)	0.016	13.7 (7.6)	13.7 (7.6)	<0.001
Cancer		39 (15.3)	331 (35.3)	0.492	33.5 (18.6)	33.5 (18.6)	<0.001
Chronic Lung Disease							
COPD		6 (2.4)	45 (4.8)	0.132	4.6 (2.6)	4.6 (2.6)	<0.001
Asthma		5 (2.0)	6 (0.6)	0.117	2.5 (1.4)	2.5 (1.4)	<0.001
Inactive Tb Scars		7 (2.7)	49 (5.2)	0.127	5.3 (2.9)	5.3 (2.9)	<0.001
Restrictive Lung Disease		2 (0.8)	8 (0.9)	0.008	1.4 (0.8)	1.4 (0.8)	<0.001
Structural Abnormality		6 (2.4)	45 (4.8)	0.132	5.0 (2.8)	5.0 (2.8)	<0.001
Intraoperative Factors							
Frequency of ABGA		1.61 (0.90)	1.87 (1.22)	0.246	1.61 (0.90)	1.93 (1.31)	0.279
Timing of ABGA		49.95 (13.32)	50.75 (15.02)	0.056	49.73 (13.43)	50.38 (14.77)	0.046
Mean Intraoperative FiO_2_		49.02 (10.84)	51.11 (12.10)	0.183	48.8 (10.92)	51.06 (12.07)	0.189
Surgery Time (min)		234.31 (97.41)	228.20 (92.99)	0.064	231.58 (94.28)	231.58 (96.40)	<0.001
Anesthesia Time (min)		231.49 (97.37)	224.87 (93.93)	0.069	228.74 (94.17)	228.74 (97.20)	<0.001
Emergency Status (E)		28 (11.0)	108 (11.5)	0.017	20.2 (11.2)	20.2 (11.2)	<0.001
Inhalation Anesthesia		248 (97.3)	905 (96.4)	0.050	175.0 (97.0)	175.0 (97.0)	<0.001
Vasopressor Use		97 (38.0)	375 (39.9)	0.039	69.4 (38.5)	69.4 (38.5)	<0.001
Intraoperative Opioids		193 (75.7)	783 (83.4)	0.192	139.6 (77.4)	139.6 (77.4)	<0.001
Use of Nerve Block		12 (4.7)	37 (3.9)	0.038	7.7 (4.3)	7.7 (4.3)	<0.001
PCA Used		198 (77.6)	747 (79.6)	0.046	141.0 (78.2)	141.0 (78.2)	<0.001
Surgery Position (%)	Fracture table	2 (0.8)	8 (0.9)	0.303	1.4 (0.8)	1.4 (0.8)	<0.001
	Lithotomy	93 (36.5)	247 (26.3)		60.4 (33.5)	60.4 (33.5)	
	Lt.LDP	11 (4.3)	67 (7.1)		9.1 (5.1)	9.1 (5.1)	
	Prone	15 (5.9)	103 (11.0)		12.5 (6.9)	12.5 (6.9)	
	Rt.LDP	18 (7.1)	50 (5.3)		13.3 (7.4)	13.3 (7.4)	
	Sitting	1 (0.4)	1 (0.1)		0.4 (0.2)	0.4 (0.2)	
	Supine	115 (45.1)	463 (49.3)		83.3 (46.2)	83.3 (46.2)	
Surgery Department (%)	GS	115 (45.1)	450 (47.9)	0.295	82.6 (45.8)	82.6 (45.8)	<0.001
	NS	28 (11.0)	153 (16.3)		22.3 (12.3)	22.3 (12.3)	
	OBGYN	27 (10.6)	66 (7.0)		18.1 (10.0)	18.1 (10.0)	
	OL	9 (3.5)	20 (2.1)		5.4 (3.0)	5.4 (3.0)	
	OS	22 (8.6)	80 (8.5)		15.9 (8.8)	15.9 (8.8)	
	OPH	1 (0.4)	1 (0.1)		0.5 (0.3)	0.5 (0.3)	
	PS	0 (0.0)	1 (0.1)		0.0 (0.0)	0.0 (0.0)	
	CS	23 (9.0)	107 (11.4)		17.9 (9.9)	17.9 (9.9)	
	URO	30 (11.8)	61 (6.5)		17.7 (9.8)	17.7 (9.8)	

Data are presented as the mean (standard deviation) for continuous variables and as a number (weighted percentage) for categorical variables. All standardized mean differences (SMDs) in the overlap-weighted cohort were <0.1, indicating an excellent balance between the groups. BMI, body mass index; ASA, American Society of Anesthesiologists; CKD, chronic kidney disease; PCA, patient-controlled analgesia; LDP, lateral decubitus position; GS, general surgery; NS, neurosurgery; OBGYN, obstetrics and gynecology; OL, otolaryngology; OPH, ophthalmology; PS, plastic surgery; CS, cardiothoracic surgery; URO, urology.

**Table 2:** Postoperative analgesic requirements and pain intensity.

Outcome Variable (MME)	Lower PaO_2_ (Mean ± SE)	Higher PaO_2_ (Mean ± SE)	Unadjusted *p* Value ^†^	Adjusted Estimate	95% CI	Adjusted *p* Value ^‡^
Strong Opioids (0–24 h, MME)	1.18 ± 0.13	1.06 ± 0.09	0.454	−0.119	−0.404 to 0.167	0.414
Strong Opioids (24–48 h, MME)	0.50 ± 0.11	0.22 ± 0.04	0.022	−0.278	−0.499 to −0.058	0.014
Strong Opioids (Total, MME)	1.68 ± 0.19	1.28 ± 0.10	0.062	−0.397	−0.771 to −0.023	0.037
Tramadol (0–24 h, MME)	1.20 ± 0.20	1.30 ± 0.13	0.691	0.093	−0.348 to 0.535	0.679
Tramadol (24–48 h, MME)	1.04 ± 0.19	1.13 ± 0.12	0.698	0.088	−0.336 to 0.512	0.684
Tramadol (Total, MME)	2.24 ± 0.35	2.43 ± 0.20	0.648	0.181	−0.561 to 0.923	0.632
Total MME (0–24 h)	2.38 ± 0.25	2.36 ± 0.16	0.931	−0.026	−0.573 to 0.522	0.927
Total MME (24–48 h)	1.54 ± 0.22	1.35 ± 0.13	0.463	−0.190	−0.667 to 0.286	0.433
Total MME (Total)	3.92 ± 0.40	3.71 ± 0.24	0.646	−0.216	−1.070 to 0.639	0.620
NRS at 0–24 h	3.53 ± 0.08	3.56 ± 0.05	0.754	0.030	−0.148 to 0.207	0.742
NRS at 24–48 h	3.35 ± 0.07	3.40 ± 0.04	0.542	0.052	−0.099 to 0.202	0.500
Maximum NRS (0–48 h)	3.43 ± 0.07	3.45 ± 0.04	0.733	0.025	−0.117 to 0.168	0.727
Frequency of Rescue Analgesics (0–48 h)	1.35 ± 0.11	1.04 ± 0.06	0.012	−0.312	−0.536 to −0.087	0.007
AAP	0 [0-0]	0 [0-0]	0.257	13.5	−9.361 to 36.320	0.246
NSAID	0 [0-0]	0 [0-0]	0.456	1.6	−2.452 to 5.657	0.438

Data are presented as weighted mean ± standard error unless otherwise specified. ^†^ *p* values were calculated using the weighted *t*-test. ^‡^ *p* values were calculated using multivariable weighted linear regression models, adjusted for age, sex, BMI, ASA physical status, surgical department, surgery time, emergency status, PCA use, nerve block, intraoperative opioid use, and comorbidities. MME, morphine milligram equivalent; NRS, numerical rating score; AAP, acetaminophen; NSAID, non-steroidal anti-inflammatory drug.

**Table 3:** Adverse outcomes.

Outcome Variable	Lower PaO_2_ (*n* = 180.3)	Higher PaO_2_ (*n* = 180.3)	Unadjusted *p* Value ^†^	Adjusted OR (95% CI)	Adjusted *p* Value ^‡^
Total PPC	47.8 (26.5)	49.6 (27.5)	0.767	1.070(0.748–1.530)	0.711
PPC within 48 h	29.2 (16.2)	34.0 (18.8)	0.355	1.252(0.831–1.887)	0.282
PPC late	34.6 (19.2)	32.4 (18.0)	0.673	0.908(0.607–1.356)	0.635
Moderate–severe PPC within 48 h	0.8 (0.4)	0.6 (0.3)	0.770	N/A	N/A
Moderate–severe PPC late	0.8 (0.5)	0.7 (0.4)	0.866	76.604 (6.374–920.612)	N/A
Total AE	18.5(10.2)	15.1 (8.4)	0.381	0.798(0.466–1.367)	0.411
Early AE	11 (6.1)	9.7 (5.4)	0.687	0.923(0.461–1.848)	0.821
Late AE	14.1 (7.8)	11.5 (6.4)	0.438	0.839(0.452–1.558)	0.578
Mortality	0.0 (0.0)	1.0 (0.5)	0.068	N/A	N/A

Data are presented as a number (%). ^†^ *p* values were calculated using the weighted Rao–Scott chi-square test. ^‡^ *p* values were calculated using multivariable weighted linear regression models, adjusted for age, sex, BMI, ASA physical status, surgical department, surgery time, emergency status, PCA use, nerve block, intraoperative opioid use, and comorbidities (cancer, stroke, and various chronic lung diseases). PPC, postoperative pulmonary complication; AE, adverse event; OR, odds ratio; CI, confidence interval; N/A, not applicable due to zero events in the control group.

**Table 4:** Sensitivity analysis of postoperative outcomes according to intraoperative PAO_2_ intensity stratification.

Outcome	Lower Higher PaO_2_ (*n* = 255)	Moderately Higher PaO_2_ (*n* = 781)	Severely Higher PaO_2_ (*n* = 158)	*P* Trend/*p* Value
Analgesic efficacy				
24–48 h strong opioid (MME)	0.50 ± 0.11	0.23 ± 0.04	0.20 ± 0.09	0.014 ^†^
Adjusted estimate (95% CI)	References	−0.281 (0.508 to −0.054)	−0.265 (−0.518 to −0.013)	0.018 ^‡^
Safety outcomes				
Total PPCs (%)	26.5	27.0	29.7	0.813 ^§^
Total adverse events (%)	10.2	8.4	8.2	0.557 ^§^

Data are presented as overlap-weighted mean ± standard error or overlap-weighted percentage. PaO_2_ intensity was defined by the maximum. ^†^ P trend was calculated using a multivariable weighted linear regression model by treating PAO_2_ intensity as an ordinal variable. ^‡^ Sensitivity analysis using maximum PAO_2_ as a continuous variable across the entire cohort (*p* = 0.018). ^§^ *p* value was calculated using a weighted Rao–Scott chi-square test. All models in Table 4 were adjusted for the same set of covariates used in Table 3. MME, morphine milligram equivalent; PPCs, postoperative pulmonary complications.

## Data Availability

The data underlying this article will be shared upon reasonable request to the corresponding author.
